# Awareness of radiographic guidelines for low back pain: a survey of Australian chiropractors

**DOI:** 10.1186/s12998-016-0118-7

**Published:** 2016-10-05

**Authors:** Hazel J. Jenkins

**Affiliations:** C5C347, Macquarie University, Balaclava Rd, North Ryde, New South Wales 2119 Australia

**Keywords:** Chiropractic, Radiography, Guideline compliance, Low back pain, X-rays

## Abstract

**Background:**

Chiropractors have been shown to refer for lumbar radiography in clinical scenarios inconsistent with the current clinical guidelines for low back pain. It is unknown whether this is due to lack of adherence with known guidelines or a lack of awareness of relevant guidelines. Therefore, the aim of this study is to determine Australian chiropractors’ awareness of, and reported adherence to, radiographic guidelines for low back pain. Demographic, chiropractic practice and radiographic usage characteristics will be investigated for association with poor guideline adherence.

**Methods:**

An online survey was distributed to Australian chiropractors from July to September, 2014. Survey questions assessed demographic, chiropractic practice and radiographic usage characteristics, awareness of radiographic guidelines for low back pain and the level of agreement with current guidelines. Results were analysed with descriptive statistics and logistic regression analysis.

**Results:**

There were 480 surveys completed online. Only 49.6 % (95 % confidence interval (95 % CI): 44.9, 54.4) reported awareness of radiographic guidelines for low back pain. Chiropractors reported a likelihood of referring for radiographs for low back pain: in new patients (47.6 % (95 % CI: 42.9, 52.3)); to confirm biomechanical pathologies (69.0 % (95 % CI: 64.5, 73.1)); to perform biomechanical analysis (37.5 % (95 % CI: 33.1, 42.0)); or to screen for contraindications (39.4 % (95 % CI: 35.0, 44.0)). Chiropractors agreed that radiographs for low back pain could be useful for: acute low back pain (54.0 % (95 % CI: 49.2, 58.7)); screening for contraindications (55.8 % (95 % CI: 51.0, 60.5)); or to confirm diagnosis and direct treatment (61.3 % (95 % CI: 56.5, 65.9)). Poorer adherence to current guidelines was seen if the chiropractor referred to in-house radiographic facilities, practiced a technique other than diversified technique or was unaware or unsure of current radiographic guidelines for low back pain.

**Conclusion:**

Only 50 % of Australian chiropractors report awareness of current radiographic guidelines for low back pain. A poorer awareness of guidelines is associated with an increase in the reported likelihood of use, and the perceived usefulness of radiographs for low back pain, in clinical situations that fall outside of current guidelines. Therefore, education strategies may help to increase guideline knowledge and compliance.

**Electronic supplementary material:**

The online version of this article (doi:10.1186/s12998-016-0118-7) contains supplementary material, which is available to authorized users.

## Background

The use of lumbar radiography within chiropractic clinical practice is well established as a necessary tool to aid diagnosis and direct appropriate treatment of the low back. The use of radiography is associated with risks to the patient, including risks from ionising radiation; unnecessary diagnosis leading to poorer patient outcomes or potentially unnecessary investigation or treatment; and higher costs [1]. Guidelines directing the appropriate use of lumbar radiography are necessary to increase the likelihood of clinically relevant information being obtained from the radiographs and minimise the associated risk.

Radiographic guidelines for low back pain (LBP) have been published by both the medical [2-4] and chiropractic professions [5]. Despite potential differences in LBP treatment methods used by the two professions, the published guidelines are consistent in their recommendations. Radiographs of the lumbar spine are recommended in cases of suspected serious pathology (ie. cancer, infection, inflammatory arthridity etc.) or trauma with suspected fracture or dislocation. Radiographs are not initially recommended in cases of nonspecific LBP with or without neurological symptoms. Radiographs may be indicated if the patient exhibits 4 to 6 weeks of non-response to treatment [2, 3, 5].

Despite the recommendations made in the published guidelines many chiropractors still believe that radiographs of the lumbar spine are useful in clinical scenarios outside the current guidelines. Surveys conducted by Ammendolia et. al. [6] and Walker et. al. [7] found that 63 and 68 % of respondents respectively would order lumbar radiographs outside the current clinical guidelines [6, 7]. Reasons for noncompliance with the guidelines were assessed through focus groups by Ammendolia et. al. [6] and Bussieres et. al. [8] and found that chiropractors believed lumbar radiographs were useful to detect spinal misalignment; detect and monitor degenerative change; screen for contraindications; educate patients; and for medicolegal reasons [6, 8], all of which are not consistent with current guidelines. Radiographic instruction by accredited chiropractic schools may also lack adherence to current guidelines, with 34 % of radiology instructors instructing students to consider using radiography to screen for pathology or contraindications and 25 % to provide patient reassurance [9]. In a study performed by Bussieres et. al. [10], it was found that the chiropractic school attended was one of the most influential predictors of future radiograph utilisation [10] and as such the radiographic instruction received may be important in directing radiographic guideline adherence.

There is some evidence that educating chiropractors regarding current radiographic guidelines is associated with increased guideline compliance [11, 12] or a reduction in radiographic referrals [13]. However, to establish whether this would be a useful intervention strategy amongst a specific chiropractic population, the current awareness of radiographic guidelines amongst those chiropractors should be identified.

Therefore, the aims of this study were to quantitatively assess whether Australian chiropractors reported awareness of current radiographic guidelines for LBP and whether they demonstrated adherence to the key messages in the guidelines. Finally, demographic, chiropractic practice and radiographic usage characteristics were investigated for association with poorer guideline adherence.

## Methods

### Study design

An online cross-sectional survey was conducted of Australian chiropractors. The survey was designed and implemented using the Qualtrics software. A link to the survey was distributed to Australian chiropractors through two methods: email distribution from two Australian chiropractic associations (the Chiropractors’ Association of Australia and the Chiropractic and Osteopathic College of Australasia); and within the quarterly newsletter from the national registration board for Australian Chiropractors (the Chiropractors’ Board of Australia). Only registered chiropractors were invited to partake in the survey. The survey was available from July to September, 2014. No identifying information was requested and participation was voluntary.

### Survey design

The survey collected information regarding demographics of the responding chiropractors and characteristics of clinical practice including: year and school of graduation from chiropractic training; years in clinical practice; use of in-house radiographic facilities; further education in radiographic imaging; and the primary manual technique style used in clinical practice. The primary outcome question in the survey asked respondents to state if they felt that they were aware of the current radiographic guidelines for LBP (yes/no/unsure). Respondents were also asked if they were aware (yes/no) of specific guidelines including those put out by both chiropractic [5] and medical [2, 4] professions. The secondary outcome questions asked respondents to indicate their likelihood of referring for radiographs of the low back on a four-point likert scale (never/occasionally/very often/always) and to indicate their level of agreement with statements regarding indications for low back radiographs on a 5-point likert scale (strongly disagree/disagree/neither agree or disagree/agree/strongly agree). The clinical scenarios used in the secondary outcome questions are listed in Table 1. Survey questions were adapted from previous studies performed by Ammendolia et. al [9, 12]. and the final survey was piloted on staff in the Department of Chiropractic, Macquarie University prior to distribution. The survey questions are available in Additional file 1.

**Table 1:** Secondary outcome questions

How often do you refer for x-rays of the lumbar spine for:	Indicate how well you agree with the following statements:
New patients^a^	X-ray's of the lumbar spine are indicated when a patient is nonresponsive to 4 weeks of conservative treatment for low back pain
Patients with the clinical suspicion of a traumatic injury	Routine x-rays of the lumbar spine are recommended prior to initiating spinal manipulative therapy (adjustments)^a^
Patients with the clinical suspicion of a red flag pathology (ie. tumour, infection, osteoporotic fracture etc.)	X-ray's of the lumbar spine are indicated to perform radiographic biomechanical analysis to assess spinal misalignments (subluxations) and to obtain spinal listings or other biomechanical information which are used to direct treatment^a^
Patients with the clinical suspicion of an inflammatory arthridity (ie. ankylosing spondylitis, rheumatoid arthritis etc.)	There is a role for the use of lumbar spine x-rays in the evaluation of patients with acute low back pain (less than one month duration), even in the absence of red flags for serious disease^a^
Patients with the clinical suspicion of a biomechanical pathology (ie. osteoarthritis, nontraumatic spondylolisthesis etc.)^a^	There is a role for the use of lumbar spine x-rays in the evaluation of patients with chronic low back pain (greater than three months duration), even in the absence of red flags for serious disease
Biomechanical analysis of the lumbar spine (spinal listings, spinal curve measurement etc.)^a^	There is a role for full spinal x-rays in chiropractic practice (other than for patients with scoliosis)
Screening for subclinical contraindications to treatment (ie. congenital anomalies, unsuspected pathology etc.)^a^	There is overutilisation of plain film x-rays in chiropractic practice in our community
Patients without low back pain as a component of a full spine x-ray series^a^	There is a role for x-rays of the lumbar spine when there are neurological signs associated with low back pain^a^
Patient reassurance or at patient request	X-rays of the lumbar spine are useful in the diagnostic work up of patients with sciatica^a^
	X-rays of the lumbar spine are useful in the diagnostic work up of patients with suspected pathology
	X-rays of the lumbar spine are useful to confirm the diagnosis and to direct appropriate treatment of low back pain^a^
	There is a role for the use of x-rays as a screening tool to find contraindications to manipulation in patients with low back pain^a^
	I am likely to refer low back pain patients for x-rays of the lumbar spine because patients often expect me to do so

^a^Outcome questions not consistent with current radiographic guidelines and used for multivariate logistic regression analysis

### Data analysis

Survey response rate was calculated as the number of completed surveys divided by the number of chiropractors sent the survey link. The survey link was sent to all registered chiropractors in Australia through the quarterly registration board newsletter, therefore the denominator used was the total number of registered chiropractors in Australia.

Descriptive statistics were used to assess the frequency of response to each of the three responses to the primary survey question regarding awareness of radiographic guidelines. Stratification of the responses to the primary research question was performed according to institute of graduation and association was tested for using the chi-squared test. Results for the secondary outcome questions were dichotomised: for those regarding the likelihood of referring for radiographs into those likely to refer for radiographs (very often/always) compared to the other responses; and for those assessing agreement with indications for referring for radiographs into those agreeing with the statement (agree/strongly agree) compared to all other responses. All of these were calculated as percentages with 95 % confidence intervals (95 % CI).

Multivariate logistic regression analysis was used to assess for association and to adjust for possible confounders between six preselected predictor variables and twelve outcome questions: five questions from the respondents’ likelihood for referring for radiographs and seven from their agreement with statements regarding the usefulness of radiographs for LBP (see Table 1). The predictor variables were: year of graduation; new graduate; history of further radiographic education; practicing techniques other than diversified technique as their primary manual technique style; referral to in-house radiographic facilities; and reported lack of awareness or unsure of current radiographic guidelines for LBP. New graduate was dichotomised to those with 5 years or less of clinical practice experience and those with more. Primary manual technique style was dichotomised into those that practiced techniques other than diversified and those that practiced diversified technique. Lack of awareness of guidelines was dichotomised to those that reported no awareness or that they were unsure and those that reported awareness. Twelve separate models were built for each of the outcome questions by entering all six predictor variables into each model. Odds ratios with 95 % CI were calculated for each variable. Multicollinearity of the six predictor variables was assessed using a correlation matrix.

## Results

### Survey respondents

On closure of the survey 480 completed responses were received. At the time of the survey there were 4859 chiropractors registered with the Chiropractic Board of Australia [14] giving a response rate of 9.9 %.

The demographic and clinical practice characteristics of respondents are shown in Table 2. The majority of respondents practiced in New South Wales (40.8 %), refer radiographs to medical radiographic facilities (74.8 %) and practice diversified technique as their primary chiropractic technique (61.4 %). The number of years in clinical practice demonstrated a good spread of respondents with the most having practiced for less than 6 years (22.9 %) and the least for more than 40 years (2.2 %).

**Table 2:** Respondent demographic and clinical practice characteristics

	Number of respondents agreeing/Total respondents answering question (%)
Australian state of clinical practice:	
NSW	196/480 (40.8)
VIC	88/480 (18.3)
SA	42/480 (8.8)
QLD	77/480 (16)
WA	46/480 (9.6)
TAS	5/480 (1.0)
ACT	11/480 (2.3)
NT	1/480 (0.2)
Outside Australia	5/480 (1.0)
Institute of graduation	
Macquarie University	185/480 (39.4)
Royal Melbourne Institute of Technology (RMIT)	130/480 (27.7)
Murdoch University	27/480 (5.8)
Other Australian institutes	54/480 (11.5)
Institutes outside Australia	73/480 (15.6)
Years in clinical practice:	
Less than 6 years	106/463 (22.9)
6–10 years	62/463 (13.4)
11–15 years	64/463 (13.8)
16–20 years	70/463 (15.1)
21–30 years	93/463 (20.1)
31–40 years	58/463 (12.5)
More than 40 years	10/463 (2.2)
Radiographic referrals:	
In-house radiographic facilities	114/469 (24.3)
Other chiropractic radiographic facilities	1/469 (0.2)
Medical radiographic facilities	351/469 (74.8)
No referral (Not currently practicing)	3/469 (0.6)
Main chiropractic technique used in clinical practice:	
Diversified technique	288/469 (61.4)
Gonstead technique	40/469 (8.5)
Activator technique	17/469 (3.6)
Thompson technique	28/469 (6.0)
Sacrooccipital technique	33/469 (7.0)
Applied kinesiology	18/469 (3.8)
Chiropractic biophysics	12/469 (2.6)
Advanced biostructural correction	8/469 (1.7)
Other technique	25/469 (5.3)

### Awareness of, and adherence to, current radiographic guidelines for low back pain

The primary outcome question asked respondents to state their awareness of current radiographic guidelines for LBP. Of 415 respondents to this question, only 206 (49.6 % (95 % CI: 44.9, 54.4)) reported definite awareness of current guidelines whereas 170 (41.0 % (95 % CI: 36.3, 45.8)) were unsure of their awareness and 39 (9.4 % (95 % CI: 7.0, 12.6)) reported that they were unaware of current guidelines. When questioned regarding specific guidelines only 68 out of 477 respondents (14.3 % (95 % CI: 11.4, 17.7)) were aware of the chiropractic guidelines written by Bussieres et. al. [5], 48 out of 479 respondents (10.0 % (95 % CI: 7.6, 13.0)) were aware of the medical guidelines written by Chou et. al. [2] and 108 out of 477 respondents (22.6 % (95 % CI: 19.1, 26.6)) were aware of the medical guidelines distributed by the National Health and Medical Research Council (NHMRC) in Australia [4].

**Fig. 1:**
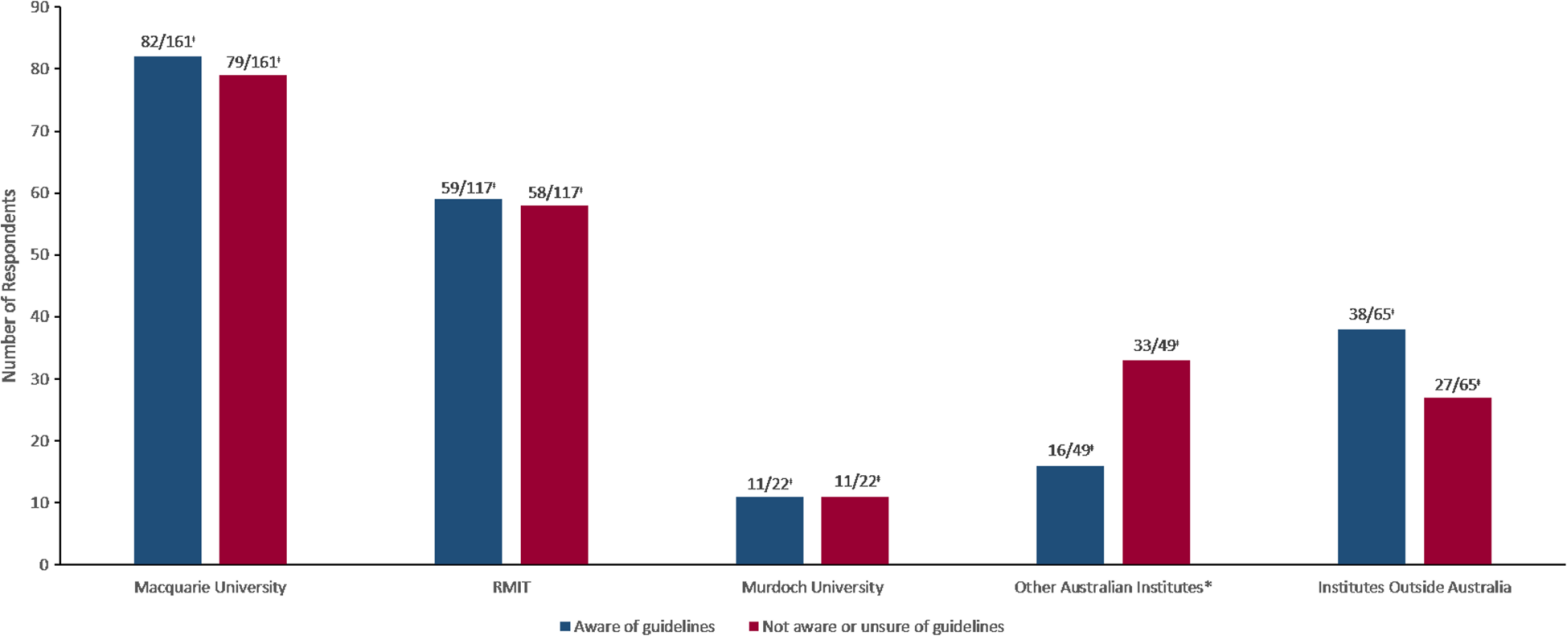
Number of respondents reporting awareness of the guidelines, stratified by institute of graduation. Key: *Other Australian institutions includes all older institutes that have now ceased operation. ^≠^ ‘Number of respondents’/’Total number of respondents answering the question’

Stratification of respondents reported awareness of radiographic guidelines for low back pain by institute of graduation is shown in Fig. 1. No significant association between the institute of graduation and the reported awareness of guidelines was found (χ^2^(8) = 14.2, *p* = 0.1).

**Fig. 2:**
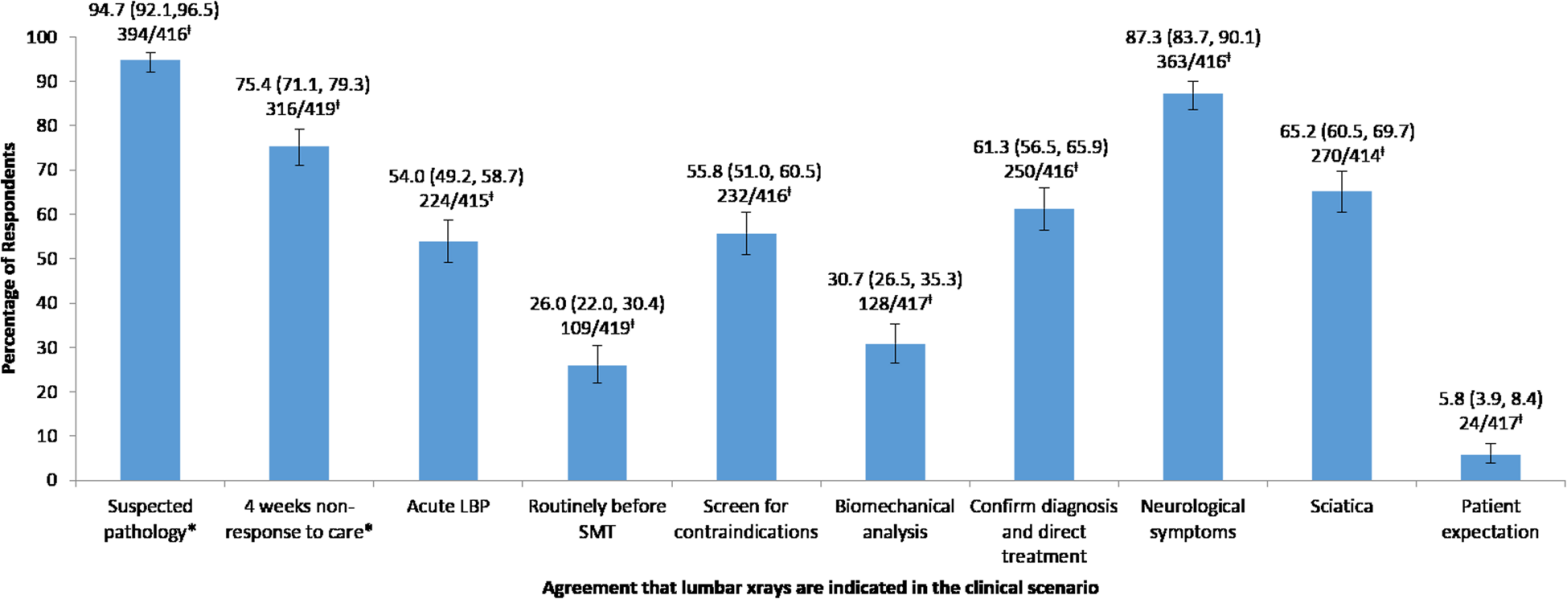
Percentage of respondents reporting a likelihood of referring for radiographs of the low back in the given clinical scenarios. Key: *Clinical scenarios consistent with current radiographic guidelines for LBP. ^≠^ Percentage with 95 % confidence intervals on top line; ‘Number of respondents’/’Total number of respondents answering the question’ on bottom line

**Fig. 3:**
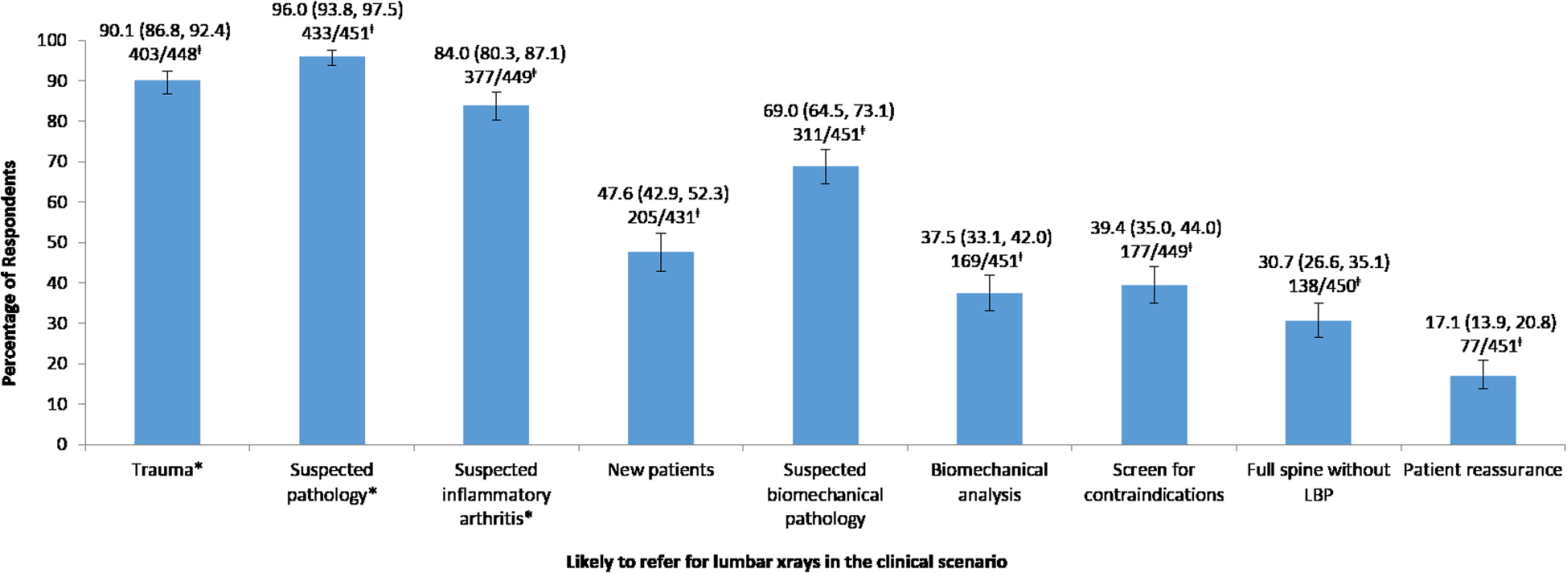
Percentage of respondents reporting agreement that radiographs of the low back may be indicated in the given clinical scenarios. Key: *Clinical scenarios consistent with current radiographic guidelines for LBP. ^≠^ Percentage with 95 % confidence intervals on top line; ‘Number of respondents’/’Total number of respondents answering the question’ on bottom line

The responses for the secondary outcome questions are depicted in Figs. 2 and 3. There was both a high likelihood of referring for radiographs, and a high agreement that radiographs were important, in clinical scenarios that adhered to current radiographic guidelines for LBP. These scenarios included suspected pathology or inflammatory arthridity, suspected trauma or non-response to care after 4 weeks. A high likelihood of referring for radiographs was also found in scenarios with poor adherence to current radiographic guidelines, including for suspected biomechanical pathology (69.0 % (95 % CI: 64.5, 73.1)). Likewise, agreement that radiographs could be useful in scenarios outside the current radiographic guidelines was also high. In particular, in cases of associated neurological symptoms (87.3 % (95 % CI: 83.7, 90.1)) or sciatica (65.2 % (95 % CI: 60.5, 69.7)).

### Factors associated with poorer adherence to current radiographic guidelines for low back pain

Analysis of associations with outcome questions showing poorer adherence to current radiographic guidelines for LBP are shown in Table 3. Year of graduation, whether or not a respondent is a new graduate or whether they had undertaken further radiographic education did not have statistically significant associations with the outcome questions. Performing in-house radiographic referrals, performing techniques other than diversified as the primary technique used or reporting either a lack of awareness or uncertainty of current radiographic guidelines showed positively correlated, statistically significant associations with the majority of the outcome questions, indicating an increased likelihood of agreeing with the outcome questions.

**Table 3:** Factors associated with statements inconsistent with current radiographic guidelines for LBP

Outcome Questions	Predictor Variables					
	Year of graduation	New graduate	Further radiographic education	Technique other than diversified	In-house radiographic referrals	Lack of awareness or unsure of radiographic guidelines
Likely to refer for radiographs for LBP:						
New patients	1.01 (0.98, 1.04)	0.98 (0.48, 2.02)	1.62 (0.98, 2.78)	2.35 (1.46, 3.79)^*^	7.23 (3.86, 13.52)^**^	1.79 (1.11, 2.90)^*^
Suspected biomechanical pathology	1.00 (0.96, 1.02)	1.66 (0.82, 3.37)	1.36 (0.80, 2.32)	1.62 (0.99, 2.68)	4.50 (2.18, 9.27)^**^	1.81 (1.13, 2.90)^*^
Biomechanical analysis	0.99 (0.96, 1.01)	1.28 (0.62, 2.61)	1.55 (0.92, 2.62)	2.21 (1.39, 3.51)^*^	4.44 (2.59, 7.62)^**^	1.72 (1.08, 2.76)^*^
Screen for contraindications	0.99 (0.96, 1.01)	1.26 (0.62, 2.54)	1.42 (0.85, 2.36)	2.30 (1.46, 3.63)^*^	3.21 (1.90, 5.42)^**^	1.08 (0.69, 1.70)
Full spine without LBP	1.00 (0.97, 1.03)	1.38 (0.65, 2.92)	1.42 (0.82, 2.46)	2.77 (1.72, 4.48)^*^	3.58 (2.09, 6.11)^**^	1.55 (0.95, 2.52)
Agree that LBP radiographs may be useful:						
Routinely before spinal manipulative therapy	1.00 (0.97, 1.04)	0.85 (0.37, 1.96)	1.33 (0.73, 2.42)	2.70 (1.60, 4.55)^*^	6.26 (3.54, 11.06)^**^	2.14 (1.24, 3.70)^*^
Biomechanical analysis	1.00 (0.97, 1.03)	1.20 (0.54, 2.67)	2.09 (1.18, 3.71)^**^	2.29 (1.38, 3.78)^*^	5.65 (3.26, 9.79)^**^	1.33 (0.80, 2.21)
Acute LBP	1.01 (0.98, 1.04)	0.45 (0.23, 0.90)^*^	1.28 (0.77, 2.13)	1.80 (1.13, 2.86)^*^	4.17 (2.28, 7.61)^**^	1.80 (1.14, 2.82)^*^
Neurological symptoms	1.00 (0.97, 1.04)	1.31 (0.51, 3.37)	1.22 (0.60, 2.48)	1.85 (0.91, 3.76)	2.31 (0.92, 5.80)	2.11 (1.12, 4.00)^*^
Sciatica	1.00 (0.97, 1.02)	0.74 (0.37, 1.49)	1.53 (0.89, 2.63)	1.22 (0.75, 1.98)	4.99 (2.46, 10.10)^**^	2.35 (1.47, 3.77)^*^
Confirm diagnosis and direct treatment	1.02 (1.00, 1.04)	0.73 (0.38, 1.44)	2.07 (1.24, 3.48)^**^	0.94 (0.59, 1.50)	4.18 (2.21, 7.92)^**^	1.35 (0.86, 2.11)
Screen for contraindications	0.99 (0.96, 1.02)	1.14 (0.58, 2.26)	1.22 (0.73, 2.05)	2.12 (1.32, 3.39)^*^	5.47 (2.86, 10.43)^**^	1.77 (1.13, 2.79)^*^

^*^Statistically significant (*p* < 0.05) with a negative association (odds ratio < 1)

^**^Statistically significant (*p* < 0.05) with a positive association (odds ratio > 1)

The correlation coefficients of the independent variables were assessed. The highest correlation coefficient found was 0.37 between further radiographic education and the year of graduation, indicating that multicollinearity is not significant for this analysis.

## Discussion

The primary finding of this study was that 49.6 % (95 % CI: 44.9, 54.4) of Australian chiropractors’ report definite awareness of current radiographic guidelines for the low back and only 14.3 % (95 % CI: 11.4, 17.7) were aware of published guidelines for the chiropractic profession. This low awareness of current guidelines is consistent with the lack of adherence found in both chiropractors’ reported likelihood of referring for radiographs and their agreement with scenarios where radiographs could be useful. Poorer reported guideline adherence was associated with chiropractors who: refer to in-house radiographic facilities; practice techniques other than diversified; or are unsure or unaware of current radiographic guidelines.

The results of this study indicate that adherence to radiographic guidelines for LBP by Australian chiropractors needs to be improved. Current guidelines recommend that radiographs are not useful for the majority of acute LBP cases, including those associated with neurological symptoms; to screen for contraindications; or to analyse for biomechanical change. Radiographs are indicated by the suspicion of trauma or pathology as a cause of LBP or after a period of four to 6 weeks non-response to treatment [5]. Although reported adherence to guidelines was high in clinical scenarios consistent with current guidelines, many chiropractors also reported that radiographs would be useful in cases of acute LBP, LBP with neurological symptoms, to screen for contraindications or to diagnose or analyse biomechanical changes, all of which show poor guideline adherence. It is important that the clinical benefit of taking a radiograph outweighs the inherent risks involved. Indications for referring for radiographs of the lumbar spine in clinical guidelines are based on best current evidence to maximise the clinical benefit to risk ratio. Poor adherence to clinical guidelines results in an increased risk to the patient as there is a decreased likelihood of clinical benefit but the same inherent risks associated with radiography. Furthermore, a lack of adherence to evidence- based clinical guidelines reflects poorly across the chiropractic profession as primary contact healthcare practitioners. If determined significant enough, this lack of adherence may result in legislative changes limiting radiographic referral rights, to improve evidence-based care and reduce unnecessary radiographs.

Adherence to radiographic guidelines may be limited by a lack of awareness of the current guidelines. Low awareness of the current guidelines in Australian chiropractors was seen in this study, particularly of those guidelines published for the chiropractic profession specifically [5]. Although an awareness of guidelines does not necessarily mean guideline messages will be adhered to, chiropractors need to be aware of the current standards they should be meeting. In addition, the odds of being adherent to the guidelines were between 1.72 to 2.35 times greater if the chiropractor reported guideline awareness in this study. Guideline education, such as online dissemination or reminders, didactic workshops and media campaigns have shown some previous evidence of effectiveness in improving guideline adherence or reducing inappropriate imaging of the lumbar spine within chiropractic populations [11-13] and may be cost-effective interventions to increase guideline awareness. Clinical scenarios with lower reported adherence to guidelines should be targeted during these educational strategies, with a focus on explaining the limited clinical benefit of referring for radiographs in these scenarios. In this study, the chiropractic institute of graduation was not significantly associated with awareness of current guidelines and the time since graduation was not significantly associated with guideline adherence. This indicates that similar proportions of chiropractors, graduating from different institutes and at different time points, have similar guideline awareness and adherence respectively. Therefore, educational strategies should be targeted both at practicing chiropractors and within chiropractic teaching institutions.

Poorer guideline adherence was associated with chiropractors referring to in-house radiographic facilities and those practicing techniques other than diversified as their primary manual technique style. Therefore, it may be important to target educational strategies towards these groups of chiropractors to improve adherence to guideline recommendations. Referral to in-house radiographic facilities showed the strongest association with poorer guideline adherence. In chiropractors referring to their own radiographic facilities the odds of being likely to take radiographs of new patients were increased 7.23 times and the odds of agreeing that radiography is useful routinely before spinal manipulative therapy were increased 6.26 times. These findings are of concern since self-referral to practitioner-owned radiographic facilities has the potential to be misused for financial incentives. Strong consideration needs to be given by these practitioners regarding the clinical justification of referring for radiographs, particularly when not in adherence with clinical guidelines. Given their responsibility in owning and operating radiographic facilities and their need to comply with national regulations for ionising radiation, strategies to increase guideline adherence within this group should be prioritised. Diversified technique is the most common spinal manipulative technique practiced among chiropractors [15-17], however, there are many other technique systems that may be used in chiropractic practice [18]. Historically, chiropractors analysed radiographs to assess for spinal alignment and guide appropriate management [19]. Although this approach is no longer recommended by radiographic guidelines [5] and is not a component of diversified technique, some chiropractic technique systems still include the use of radiographic analysis as a diagnostic tool to detect biomechanical changes [18, 20, 21]. Two of these technique systems, Gonstead technique and Chiropractic Biophysics, were the primary technique system used by 10.2 % of respondents, representing more than one quarter of respondents who did not practice diversified technique. The inclusion of techniques emphasising radiography as a diagnostic tool may explain the poorer adherence to guidelines in chiropractors practicing techniques other than diversified technique. Consideration needs to be given by chiropractors practicing these techniques to determine whether radiographic analysis gives sufficient clinical benefit to justify the associated risks.

The awareness of radiographic guidelines for low back pain within Australian chiropractors has not been previously assessed in the literature. Similarly, no studies assessing awareness of guidelines in other chiropractic populations were found. This information is important as this may indicate the need for education of the key guideline messages to the chiropractic population. Three previous studies have quantitatively assessed the adherence of registered chiropractors to radiographic guidelines for the management of LBP. Two surveys performed in Canada with 26 and 32 responses respectively found that 63 and 59 % would use radiography for acute LBP without indicators of potential pathology and 68 and 66 % thought that radiography was useful in the evaluation of acute LBP [6, 12]. Despite the small sample sizes the results are consistent with those of this study. Similarly, Walker et. al. [7] surveyed 274 Australian chiropractors and found that 68 % of respondents would order radiographs in clinical situations where it was not indicated [7]. Previous qualitative research has assessed reasons for poor adherence with guidelines and found that reasons for taking radiographs for LBP include to perform biomechanical assessment, to screen for contraindications, to confirm a diagnosis or direct treatment, to educate patients, for medicolegal reasons or for financial incentives [6, 8]. The results of this study provide quantitative evidence supporting these findings, with respondents agreeing that radiographs of the low back are useful to screen for contraindications (55.8 %), to confirm a diagnosis or direct treatment (61.3 %) and for biomechanical analysis (30.7 %). In the current study, institute of graduation was not significantly associated with awareness of current guidelines for LBP radiography. In contrast, an American study found that the chiropractic school attended was strongly associated with rates of imaging utilisation [10]. There are only three currently operating institutes in Australia with graduate chiropractors compared to 21 American institutes in the study by Bussieres et. al. [10], giving a wider range for variation in American compared to Australian institutes. This variation, the different geographic locations, or the different outcomes measures may account for the differences in study findings.

Key strengths of this study include the primary outcome question and the analysis for potential associations with poorer reported guideline adherence. Previous studies have shown chiropractors’ to demonstrate a lack of adherence to radiographic guidelines for LBP [6-8], however, they have not assessed whether chiropractors are aware of current radiographic guidelines. This may impact potential education strategies to increase guideline adherence. Knowledge of potential associations with poorer guideline adherence may also help direct future strategies to improve guideline adherence. Performing multivariate logistic regression analysis enabled assessment for these associations while accounting for potential confounders.

The main limitation of this study is the inability to calculate an accurate response rate. The response rate was calculated as 9.9 % based upon the number of completed surveys compared to the total number of registered chiropractors in Australia. However, this is likely to be an underestimate as the number of people who actually received the survey link could not be accurately determined. The survey was distributed via email distribution to association members and a link in the quarterly newsletter from the Chiropractors’ Board of Australia. Although all registered Australian chiropractors should have received the survey information, it is unknown how many actually received and opened the emails or newsletter and how many followed the link to obtain information about the survey. Calculation of an accurate response rate is necessary to assess non-response bias. As this could not be achieved, assessment of representativeness of the respondent sample was addressed by comparing demographic characteristics from survey respondents to demographic data from the Chiropractic Board of Australia and previous research. Data from the Chiropractors’ Board of Australia [14] showed a similar distribution of chiropractors by Australian state or territory except for a higher number of survey respondents from New South Wales (41 % compared to 33 %) and a lower number from Victoria (18 % compared to 26 %). Diversified was listed as the primary manual technique style for 61 % of survey respondents, which is consistent with previous research [16, 17]. Likewise, less than a quarter of respondents referred to in-house radiographic facilities which is also consistent with previously published research [17, 19]. Although there are some similarities between demographics of survey respondents and previously published data we cannot eliminate the possiblity of non-response bias. The results of this survey should therefore be interpreted with caution as they may not be reflective of the Australian chiropractic population as a whole. Validated questions could not be found for the secondary outcome questions in this study. The questions used were adapted from those previously used in the literature [9, 12] and were informally piloted prior to use. The secondary outcome questions were asked in two different formats: assessing the respondents reported likelihood of referring for radiographs, and their reported agreement with the usefulness of radiographs, in certain clinical scenarios. Responses and the results of the multivariate logistic regression analysis were similar despite the different question formats used, suggesting that they measured a similar construct.

It is important that Australian chiropractors are aware of current guidelines and demonstrate increased adherence to those guidelines. Implementation of guideline education strategies, both within chiropractic teaching institutes and to chiropractors within the profession, should be undertaken and may be a low-cost strategy to increase awareness and adherence to current guidelines. Further research should be carried out to measure the effectiveness of these educational strategies and whether further intervention is necessary. Targeted interventions may be needed in specific groups within the chiropractic profession, including those with in-house radiographic facilities and those practicing techniques other than diversified technique. Further research may be needed to identify the barriers specific to these populations and design suitable implementation interventions accordingly.

## Conclusion

This study indicates that only 49.6 % of Australian chiropractors are aware of current radiographic guidelines for LBP. This lack of awareness is reflected in a high percentage of respondents agreeing that radiographs would be useful and that they are likely to refer for radiographs in clinical scenarios that do not adhere to current guidelines. Poorer adherence to current radiographic guidelines was associated with chiropractors who referred to in-house radiographic facilities; practiced techniques other than diversified; or those who reported a lack of awareness of the current guidelines. These findings may be useful in designing education strategies to help improve guideline awareness and guideline adherent behaviour amongst Australian chiropractors.

## Additional file

Additional file 1: Survey questions. (PDF 360 kb)

## Abbreviations

95 % CI: 95 % Confidence Interval
LBP: Low Back Pain
NHMRC: National Health and Medical Research Council
