# Supplementary material for: Awareness of radiographic guidelines for low back pain: a survey of Australian chiropractors
Source: Chiropr Man Therap. 2016 Oct 5;24:39. doi: 10.1186/s12998-016-0118-7 (PMC5051064; doi:10.1186/s12998-016-0118-7)
Supplement: Additional file 1: — Survey questions. (PDF 360 kb) [file 12998_2016_118_MOESM1_ESM.pdf]

Default Question Block

Where did you receive the link to this survey from?

- AHPRA Chiropractic Board of Australia quarterly newsletter
- Email from the Chiropractors Association of Australia (CAA)
- Email from the Chiropractic and Osteopathic College of Australia (COCA)
- Facebook
- Other:

Supervisor's name: Hazel Jenkins
Supervisor's title: BMedSci, MChiro, MAppSci; Lecturer, Department of Chiropractic, Macquarie University

Co-Investigator: Sean Gilena; Student MChiro, Macquarie University
Co-Investigator: Loukia Papageorgiou; Student MChiro, Macquarie University
Co-Investigator: Melinda Tracey; Student MChiro, Macquarie University
Co-Investigator: Jaselle van Gestel; Student MChiro, Macquarie University

Participant Information and Consent

Name of Project: X-ray usage and compliance with guidelines by Australian chiropractors: a national survey.

You are invited to participate in a survey of Australian chiropractors. The purpose of the survey is to identify gaps in the knowledge of practicing chiropractors regarding current radiographic guidelines for low back pain and how this relates to guideline compliance.

The study is being conducted by 2nd year Masters of chiropractic students at Macquarie University as listed above to meet the requirements of the Masters of Chiropractic degree. The research is being conducted under the supervision of Mrs Hazel Jenkins of the Department of Chiropractic: Ph (02) 98509383; email hazel.jenkins@mq.edu.au.

If you decide to participate, you will be asked to answer a short survey, which consists of a series of questions. These questions detail your demographics, your understanding of x-ray guidelines and your clinical justification for x-ray recommendation.

Any information or personal details gathered in the course of the study are confidential, except as required by law. No individual will be identified in any publication of the results. This email has been distributed directly by AHPRA, with no personal details released. Each survey is completely anonymous. A summary of the results of the data can be made

available to you on request, by contacting the supervisor, Hazel Jenkins.

Participation in this study is entirely voluntary; you are not obliged to participate and if you choose not to participate you may do so without reason and without consequence. Once you complete in the survey you are unable to withdraw your responses due to the anonymous nature of the responses. If you choose not to participate please select 'no' to the question below. Completion of the survey indicates that you have consented to have the submitted data used for these research purposes. The data obtained in this research may be used for future projects and shared with other researchers, however, this will be in a non-identifiable format due to the anonymous nature of this research.

If you have any questions regarding this project, please contact the research project supervisor Hazel Jenkins on (02) 9850 9383 or email: hazel.jenkins@mq.edu.au.

The ethical aspects of this study have been approved by the Macquarie University Human Research Ethics Committee. If you have any complaints or reservations about any ethical aspect of your participation in this research, you may contact the Committee through the Director, Research Ethics (telephone (02) 9850 7854; email ethics@mq.edu.au). Any complaint you make will be treated in confidence and investigated, and you will be informed of the outcome.

Do you wish to participate in this survey and consent to having this data collected? (Please note all data collection is completely anonymous).

- Yes
- No

Are you currently in private practice?

- Yes
- No. Please go to the next page

How would you describe your work load in private practice?

- Full-time
- Part-time

How many years have you been in chiropractic clinical practice?

- <6
- 6-10
- 11-15
- 21-30
- 31-40
- >40

- 16-20

In which Australian state or territory do you practice?
You may select more than one response.

- New South Wales
- Victoria
- South Australia
- Queensland
- Western Australia
- Tasmania
- Australian Capital Territory
- Northern Territory
- I practice outside of Australia. Please enter the country you practice in.

Do you predominantly practice in a rural or a metropolitan setting?

- Metropolitan setting
- Rural setting
- I practice in both rural and metropolitan settings

What year did you finish your chiropractic studies?

At which institution did you complete your chiropractic studies?

- Macquarie University
- RMIT
- Murdoch University

- New Zealand College of Chiropractic
- Other Australian institution (Please name)
- Overseas institution (Please name institution and country)

Where do you refer your patients for x-rays?

- I use my own in-house x-ray facilities
- I refer to a colleagues chiropractic practice for x-rays
- I refer to a medical radiology practice
- I do not refer patients for x-rays

Which chiropractic technique do you mainly utilise?

- Diversified
- Gonstead
- Activator methods
- Thompson technique
- Sacrooccipital technique (SOT)
- Applied Kinesiology (AK)
- Chiropractic BioPhysics (CBP)
- Advanced Biostructural Correction (ABC)
- Other (Please name)

Please select a response that indicates how often you refer for x-rays of the lumbar spine for:

	Never	Occasionally	Very Often	Always
New patients	○	○	○	○
Patients with the clinical suspicion of a traumatic injury	○	○	○	○

Patients with the clinical suspicion of a red flag pathology (ie. tumour, infection, osteoporotic fracture etc)	○	○	○	○
Patients with the clinical suspicion of an inflammatory arthridity (ie. ankylosing spondylitis, rheumatoid arthritis etc.)	○	○	○	○
Patients with the clinical suspicion of a biomechanical pathology (ie. osteoarthritis, nontraumatic spondylolisthesis etc.)	○	○	○	○
Biomechanical analysis of the lumbar spine (spinal listings, spinal curve measurement etc.)	○	○	○	○
Screening for sub-clinical contraindications to treatment (ie. congenital anomalies, unsuspected pathology etc.)	○	○	○	○
Patients without low back pain as a component of a full-spine x-ray series	○	○	○	○
Patient reassurance or at patient request	○	○	○	○

As a chiropractor are you aware of the current radiographic guidelines for low back pain?

- Yes
- No
- Unsure

Which radiographic guideline/s are you aware of? (Please mark all answers that apply)

- Chiropractic diagnostic imaging conduct and performance: Chiropractic board of Australia (AHPRA)
- Diagnostic imaging practice guidelines for musculoskeletal complaints in adults: Bussieres et al. JMPT (2007)
- Lumbar imaging in acute non-specific low back pain: NHMRC, Australian Government
- Diagnostic imaging pathways:Department of Health, Western Australia
- Diagnosis and treatment of low back pain: a joint clinical practice guideline: Chou et al. American college of physicians and American pain society Ann Intern Med (2007)
- Other (Please name)

Have you undertaken any further education on radiographic indications or guidelines since finishing your chiropractic degree?

- Yes
- No

Please select a response that indicates how well you agree with the following statements.

	Strongly	Neither Agree nor	Strongly

	Disagree	Disagree	Disagree	Agree	Agree
X-ray's of the lumbar spine are indicated when a patient is non-responsive to 4 weeks of conservative treatment for low back pain	○	○	○	○	○
Routine x-rays of the lumbar spine are recommended prior to initiating spinal manipulative therapy (adjustments)	○	○	○	○	○
X-ray's of the lumbar spine are indicated to perform radiographic biomechanical analysis to assess spinal misalignments (subluxations) and to obtain spinal listings or other biomechanical information which are used to direct treatment	○	○	○	○	○
There is a role for the use of lumbar spine x-rays in the evaluation of patients with acute low back pain (less than one month duration), even in the absence of red flags for serious disease	○	○	○	○	○
There is a role for the use of lumbar spine x-rays in the					

	Strongly Disagree	Disagree	Neither Agree nor Disagree	Agree	Strongly Agree
evaluation of patients with chronic low back pain (greater than three months duration), even in the absence of red flags for serious disease	○	○	○	○	○

	Strongly Disagree	Disagree	Neither Agree nor Disagree	Agree	Strongly Agree
There is a role for full spinal x-rays in chiropractic practice (other than for patients with scoliosis)	○	○	○	○	○
There is overutilisation of plain film x-rays in chiropractic practice in our community	○	○	○	○	○
There is a role for x-rays of the lumbar spine when there are neurological signs associated with low back pain	○	○	○	○	○
X-rays of the lumbar spine are useful in the diagnostic work up of patients with sciatica	○	○	○	○	○
X-rays of the lumbar spine are useful in the diagnostic work up of patients with suspected pathology	○	○	○	○	○

	Strongly Disagree	Disagree	Neither Agree nor Disagree	Agree	Strongly Agree
X-rays of the lumbar spine are useful to confirm the					

diagnosis and to direct appropriate treatment of low back pain	○	○	○	○	○
There is a role for the use of x-rays as a screening tool to find contraindications to manipulation in patients with low back pain	○	○	○	○	○
I am likely to refer low back pain patients for x-rays of the lumbar spine because patients often expect me to do so	○	○	○	○	○

Please write any further comments you may have regarding this topic in the box below:

Thank you for completing this questionnaire. We value your time and appreciate that you have contributed to our research.
